# Investigation of the Association between Bilateral Selective Anterograde Cerebral Perfusion and Postoperative Ischemic Stroke in Obese Patients with Emergency Surgery for Acute Type A Aortic Dissection

**DOI:** 10.3390/medicina60040661

**Published:** 2024-04-19

**Authors:** Mircea Robu, Bogdan Radulescu, Irina Maria Margarint, Cornel Robu, Ovidiu Stiru, Andrei Iosifescu, Silvia Preda, Mihai Cacoveanu, Cristian Voica, Vlad Anton Iliescu, Horatiu Moldovan

**Affiliations:** 1 Faculty of Medicine, Carol Davila University of Medicine and Pharmacy, 050474 Bucharest, Romania; mircea.robu@drd.umfcd.ro (M.R.); cornel.robu@drd.umfcd.ro (C.R.); ovidiu.stiru@umfcd.ro (O.S.); iosifescuag@gmail.com (A.I.); silvia-mihaela.pieleanu@drd.umfcd.ro (S.P.); catalin.cacoveanu@gmail.com (M.C.); vladanton.iliescu@gmail.com (V.A.I.); horatiu.moldovan@umfcd.ro (H.M.); 2 Emergency Institute for Cardiovascular Diseases “Prof. Dr. Iliescu”, 022322 Bucharest, Romania; 3 Department of Cardiac Surgery, Emergency Clinical Hospital for Children “Maria Skłodowska Curie”, 077120 Bucharest, Romania; 4 Department of Cardiovascular Surgery, Emergency Clinical Hospital Bucharest, 014461 Bucharest, Romania; cristian.voica@drd.umfcd.ro; 5 Academy of Romanian Scientists, 050711 Bucharest, Romania

**Keywords:** dissection, thoracic aorta, ischemic stroke, cerebral perfusion, obesity

## Abstract

*Background and objectives:* The relationship between cerebral perfusion and new postoperative ischemic stroke in obese patients is not well defined. The aim of this study was to investigate the association between selective bilateral anterograde cerebral perfusion and new postoperative ischemic stroke in obese patients with emergency surgery for acute type A aortic dissection. *Materials and methods:* A total of 292 patients with emergency surgery for acute type A aortic dissection were included in this study. Patients with hemorrhagic stroke or ischemic stroke with severe neurological dysfunction at admission that were not candidates for surgery; patients who died in the first 48 h after intensive care admission and patients with incomplete medical records were excluded. *Results:* The mean age was 59.42 ± 10.68 years and the mean Euroscore was 9.12 ± 1.63. Obesity was present in 76.4%, the incidence of new postoperative ischemic stroke was 27.5%, and the postoperative mortality rate was 26.7%. The mean cardiopulmonary bypass time was 206.81 ± 75.48 min, the aortic cross-clamp time was 118.2 ± 46.42 min, and 90% of cases required cerebral perfusion. The mean cerebral perfusion time was 30.8 ± 24.41 min. Obese patients had a higher frequency of in-hospital death (*p* = 0.009), smoking (*p* = 0.036), hypertension (*p* = 0.023), left common carotid artery dissection (*p* < 0.001), right common carotid artery dissection (*p* = 0.029), femoral artery cannulation (*p* = 0.026), aortic root replacement (*p* = 0.009), aortic valve replacement (*p* = 0.005) and early reintervention for bleeding (*p* = 0.004). Using logistic regression, selective bilateral anterograde cerebral perfusion over 40 min in obese patients was independently associated with new postoperative ischemic stroke (OR = 2.35; 95%CI = 1.36–4.86; *p* = 0.021). *Conclusions:* A patient-tailored strategy for cerebral perfusion should be considered in obese patients, considering the high atheromatous burden of the supra-aortic vessels in these patients and the potential risk of atheromatous embolization associated with this technique.

## 1. Introduction

**Figure 1:**
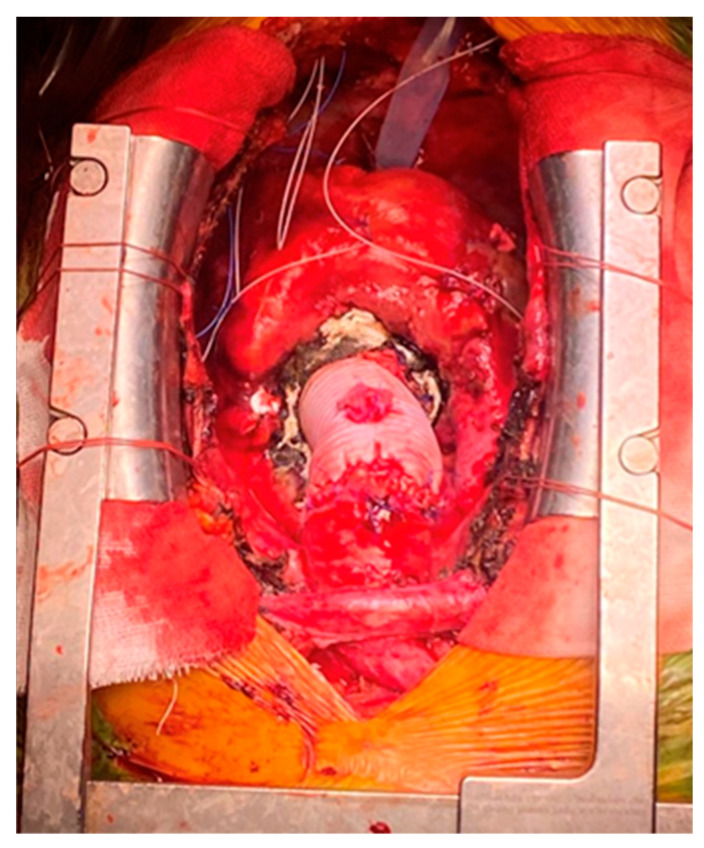
Supra-coronary ascending aorta replacement.

**Figure 2:**
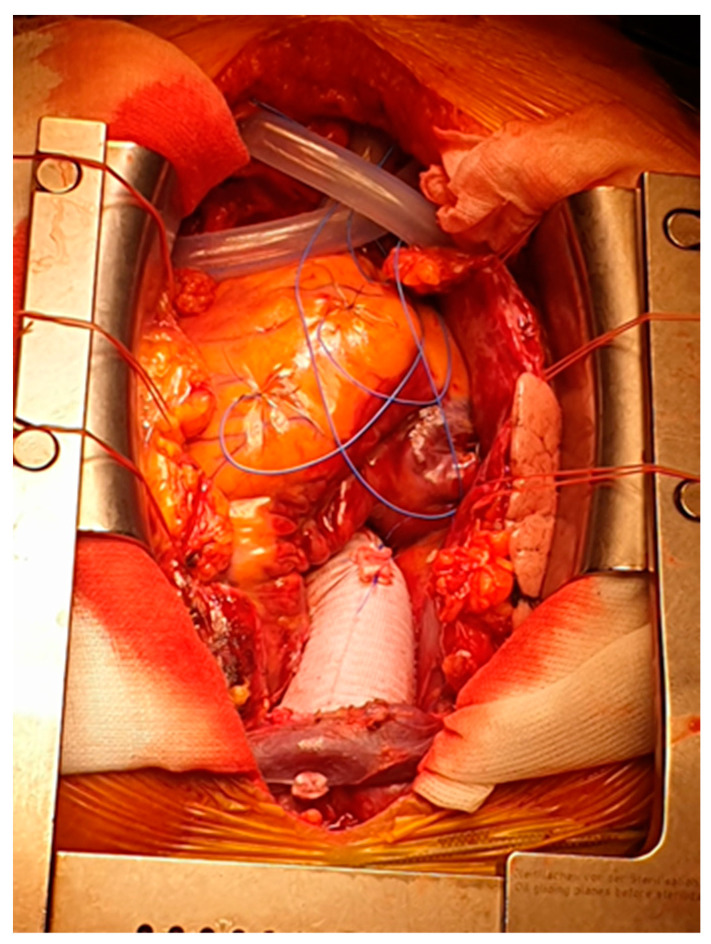
Ascending aorta and hemiarch replacement.

**Figure 3:**
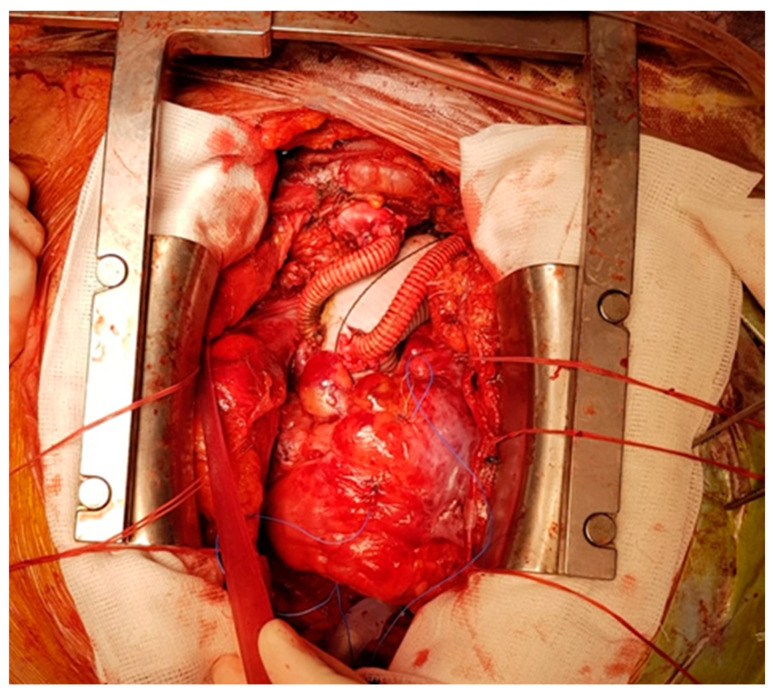
Total aortic arch replacement with a Dacron graft and separated reconnection of supra-aortic vessels.

Acute type A aortic dissection (ATAAD) is a complex cardiovascular disease, with a 5.8% mortality rate at 48 h with medical treatment alone [1,2]. Emergency central aortic surgery (Figure 1, Figure 2 and Figure 3) with the main goal of resecting the primary intimal tear and restoring flow in the true lumen is the preferred choice of treatment [3]. Complete arch replacement, a hemiarch approach, and open distal anastomosis necessitate a period of circulatory arrest. Antegrade cerebral perfusion (ACP) is the preferred strategy for brain protection during circulatory arrest and can increase the time of hypothermic circulatory arrest (HCA) [4,5]. From a technical point of view, selective ACP is performed with two cannulas inserted under direct vision into the innominate and left common carotid arteries. This technique provides superior brain protection when the HCA exceeds 40–50 min [6].

Despite advances in strategies for brain protection, the incidence of neurological complications in these patients is high and ranges from 17 to 48% [7,8,9]. One of the most dreaded neurological complications regarding mortality and morbidity is ischemic stroke, with an incidence of up to 24.8% [10]. 

The incidence of obesity is increasing in all industrialized countries and frequent comorbid conditions such as diabetes mellitus, hypertension, hyperlipidemia, and obesity-induced systemic inflammation pose a challenge to care during cardiac surgery, especially those with cardiopulmonary bypass [11]. Patients with obesity are more predisposed to developing ATAAD [12]. Moreover, obese patients with emergency surgery for ATAAD had higher operative mortality and an increased risk of low cardiac output syndrome and pulmonary complications [12,13]. 

The relationship between obesity and postoperative NC in patients with surgery for ATAAD is not well defined. This study investigated the association between obesity and postoperative ischemic stroke in patients with emergency surgery for ATAAD with HCA and selective ACP. 

## 2. Materials and Methods

Between January 2015 and May 2023, 319 patients were transferred to our center for ATTAD management. The diagnosis was based on chest computer tomography (CT) with intravenous contrast before admission. After a cardiology consult in the emergency department with mandatory transthoracic echocardiography, patients who were candidates for emergency surgery were transferred into the operating room. Clinical characteristics and demographic data were collected from medical records and the electronic health system.

Inclusion criteria: patients with acute type A aortic dissection according to the Stanford classification are considered for emergency surgery.

Exclusion criteria: (1) patients with hemorrhagic stroke documented on computed tomography (CT) scans before admission who were not candidates for surgery; (2) patients with ischemic stroke documented on computed tomography (CT) scans before admission and severe neurological dysfunction who were not candidates for surgery; (3) patients who died in the operating room or within the first 48 h after intensive care admission; (4) patients in the postoperative setting for whom the neurological status could not be evaluated; (5) patients with incomplete medical records. 

Obesity was defined as a BMI over 30 kg/m^2^.

Neurological examination and head CT were the methods used for the diagnosis of a new postoperative ischemic stroke. The Modified Rankin Scale (mRS) was the tool used for quantification of the degree of disability at discharge, based on data collected from clinical charts. 

The European System for Cardiac Operative Risk (EuroSCORE) was utilized to assess operative mortality. 

### 2.1. Surgical Technique

Cardiopulmonary bypass (CPB) was achieved with a two-stage venous cannula and axillary or femoral artery cannulation under direct vision after general anesthesia and median sternotomy. Another strategy for arterial cannulation was inserting a cannula directly into the dissected ascending aorta when the axillary or femoral arteries were not suitable, under transoesophageal echography guidance. We use cold (4 °C), crystalloid cardioplegia, which is usually administered in a retrograde manner. Venting of the left ventricle was achieved with a cannula inserted via the superior left pulmonary vein inserted through the left atrium and mitral valve. Also, the pericardial well was flooded with carbon dioxide early in the intervention to minimize the risk of air embolism. Systemic cooling of the patient to 25–28 °C was applied after CPB was started. 

The first step was performing an open distal anastomosis with circulatory arrest and BSACP. The hemiarch approach is usually the preferred method, with total arch replacement being reserved for cases where a primary entry tear cannot be excluded with this strategy. We prefer the hemiarch technique because of the higher risk of neurological complications when total arch replacement is selected [10]. Cerebral oxygenation was monitored with near-infrared spectroscopy (NIRS) in all patients. 

The BSACP protocol was the following: 13F or 15F ballon-tip cannulas were used to administer retrograde cardioplegia and were inserted into the innominate artery and left common carotid artery under direct vision. An individual pump and pressure line were used for each cannula. Perfusion parameters for a target value of 50–70% on NIRS are for the left side: pressure of 60–70 mmHg with a flow of 180–230 mL/min and for the right side: perfusion pressure of 60–70 mmHg with a flow between 150–200 mL/min. After open distal anastomosis was performed, CPB was restarted, and the supra-aortic vessels were unclamped after de-airing. During the rewarming of the patient, the proximal repair was completed. 

A special consideration should be mentioned regarding cardiac de-airing after aortic repair of dissection. A “Y” cannula generally used for anterograde cardioplegia administration in elective cases is inserted directly into the aortic prosthesis and deairing is performed via this cannula. Also, this cannula is utilized to vent the aortic root until completion of the cardiopulmonary bypass. 

### 2.2. Statistical Analysis

Wizard 2 Statistical Software for Mac OS (Wizard–Statistics & Analysis^®^, Raipur, Chattisgarh, India) was used for statistical analysis. Summary statistics are presented as absolute numbers and percentages for categorical values and as mean ± standard deviation for continuous values. 

The incidence of new postoperative ischemic stroke in patients with emergency surgery for ATAAD was investigated. Patients with a BMI over 30 kg/m^2^ were divided into two groups: patients with selective ACP below and over 40 min, considering that data from the literature suggests that ACP is safer when DHCA exceeds 40 min [6]. In order to investigate the association of selective ACP time in obese patients undergoing emergency surgery for ATAAD and the development of new postoperative ischemic stroke, multivariable analysis was performed using logistic regression and taking into account a model that included variables achieving a *p*-value < 0.5 in univariate analysis. A predictive modeling strategy with the backward stepwise method of entering data was then used. 

Logistic regression results are presented as odds ratios (OR) with confidence limits and *p*-values. *p* < 0.05 was considered statistically significant. An independent *t*-test or Chi-square test was used for comparing the neurologically complicated and uncomplicated groups.

## 3. Results

### 3.1. General Characteristics of the Study Population

A total of 292 patients with ATAAD and emergency surgery were included in the study. Of these, 27 patients were excluded (3 patients with hemorrhagic stroke and 2 patients with massive ischemic stroke that were not candidates for surgery, 4 patients with unresponsive cardiac arrest at admission, 5 patients with irreversible peripheric ischemia, 13 patients died on the operating table, 10 because of hemorrhagic shock and 3 patients had myocardial infarction). The preoperative data are summarized in Table 1. 

The mean age was 59.42 ± 10.68 years and the mean Euroscore was 9.12 ± 1.63. There was a 3.89 ± 2.37 h mean time from diagnosis of ATAAD to surgery. Obesity was present in 76.4% of cases. The most frequent complaint at admission was thoracic pain in 92.1% of patients followed by abdominal pain in 12.3% and 11% of cases presented for syncope. Preoperative ischemic stroke was documented on head CT in 7 patients (2.4%), 30 patients presented with lower leg ischemia, and mesenteric ischemia was documented based on clinical examination and contrast abdominal CT in 5 patients (2%). A bicuspid aortic valve was present in 9.24% of cases, severe acute aortic regurgitation in 25.3% of patients, and cardiac tamponade in 12.7% of cases. The innominate artery was the most frequently dissected supra-aortic artery in 19.2% of cases, followed by the left common carotid artery in 12.9% of patients. The right common carotid artery was dissected in 7.7% of cases. In 48.6% of cases, the primary entry tear was at the level of the ascending aorta. Intramural hematoma was diagnosed in 8.6% of cases and 2.1% of patients had penetrating aortic ulcers. 

Postoperative death occurred in 26.7% (78 patients). Causes of death were the following: septic shock in 36 patients (46.15%) cardiogenic shock in 12 patients (15.38%), mixed shock in 9 cases (11.53%), and hemorrhagic shock in 21 patients (26.92%). 

### 3.2. Intraoperative and Postoperative Data

Table 2 summarises the intraoperative and postoperative data. 

The incidence of a new postoperative ischemic stroke was 27.5% (81 patients). The incidence of in-hospital death in patients with ischemic stroke was 31.8% (28 patients) and 81.8% (73 patients) were obese. The mean cardiopulmonary bypass time was 206.81 ± 75.48 min and the aortic cross-clamp time was 118.2 ± 46.42 min. In 90% of cases, cerebral perfusion was applied with a mean time of 30.8 ± 24.41 min. The supracoronary ascending aorta was replaced in 96.9% of cases. At the level of the aortic arch, hemiarch replacement was performed in 45.9% of patients and total arch replacement was selected in 5.5% of cases. Root replacement was necessary for 23.3% of patients. Concomitant procedures were required in 72 patients: coronary artery bypass grafting in 5.5% of cases, mitral valve repair in 1% of cases, aortic valve replacement in 16.8% of cases, peripheral V-A ECMO in two patients, one patient required a femoral-femoral bypass and one patient had aortic coarctation repair. Axillary artery cannulation was performed in 64.5% of cases, followed by femoral artery cannulation in 31.5% of patients. Early reintervention for mediastinal bleeding was necessary in 24.1% of patients. The mean intensive care stay was 11.2 ± 13.6 days, prolonged more than 24 h mechanical ventilation was necessary in 74% of patients, and dialysis was required in 51% of cases. Postoperative mesenteric ischemia was seen in 22 patients (7.5%) while 6 patients (2.1%) developed deep sternal wound infections. Treatment for mesenteric ischemia was open abdominal surgery with resection of affected bowel in 21 patients while 1 patient received interventional treatment with stenting of the superior mesenteric artery. 

### 3.3. Characteristics of Obese Patients

Table 3 shows the comparison between the patients with and without obesity. Regarding preoperative characteristics, obese patients had a higher frequency of in-hospital death (*p* = 0.009), hyperlipidemia (*p* = 0.03), smoking (*p* = 0.036), hypertension (*p* = 0.023), left common carotid artery dissection (*p* < 0.001) and right common carotid artery dissection (*p* = 0.029). A higher frequency of femoral artery cannulation (*p* = 0.026), aortic root replacement (*p* = 0.009), aortic valve replacement (*p* = 0.005), and early reintervention for bleeding (*p* = 0.004) were observed in obese patients. No differences between the two groups were observed regarding postoperative characteristics. 

### 3.4. Characteristics of Patients with and without Ischemic Stroke 

Table 4 shows the comparison of different intraoperative parameters in patients with and without ischemic stroke. Patients with stroke had significantly higher cardiopulmonary bypass times (*p* = 0.018) and cerebral perfusion times (*p* = 0.18). No significant difference was observed in cerebral perfusion pressure and flow. Also, no difference was observed regarding the site of arterial cannulation. Table 5 presents the Modified Rankin Scale at discharge. No patient had a full recovery, while 32 patients with ischemic stroke died.

### 3.5. Logistic Regression

Variables included in the univariate analysis were the following: age, smoking, hypertension, female sex, diabetes, chronic obstructive lung disease, family history of aortic dissection, anemia, renal dysfunction, hepatic dysfunction, lactic acidosis, BMI, at admission: thoracic pain, abdominal pain, syncope, lower leg ischemia, mesenteric ischemia, cardiac tamponade, severe aortic regurgitation, severe ventricular disfunction, intimal tear in the ascending aorta, intimal tear in the aortic arch, innominate artery dissection, right common carotid artery dissection, right subclavian artery dissection, left common carotid artery dissection, left subclavian artery dissection, cardiopulmonary bypass time, aortic cross clamp time, cerebral perfusion time, femoral artery cannulation, axillary artery cannulation, aortic root replacement, supracoronary ascending aorta replacement, hemiarch replacement, total arch replacement, coronary artery bypass grafting, aortic valve replacement, mitral valve repair, and early reintervention for bleeding.

Results of the univariate analysis of variables associated with new postoperative ischemic stroke achieving a *p*-value < 0.5 are presented in Table 6. Cardiac tamponade at admission (OR = 6.27; 95%CI = 2.57–15.25; *p* < 0.01) and dissection of innominate artery (OR = 0.21; 95%CI = 0.06–0.72; *p* = 0.013) were included in the final model after backward selection. Selective ACP over 40 min in obese patients was associated with new postoperative ischemic stroke (OR = 2.18; 95%CI = 1.12–4.22; *p* = 0.021) in univariate analysis and after model adjustment was independently associated with new postoperative ischemic stroke (OR = 2.35; 95%CI = 1.14–4.86; *p* = 0.021). 

## 4. Discussion

The main findings of this study are that selective ACP over 40 min is associated with new postoperative ischemic stroke in obese patients (BMI > 30 kg/m^2^) undergoing emergency surgery for ATAAD with HCA. This result is consistent with data from the literature regarding risk factors for neurological complications (Table 7). Obese patients had a higher frequency of in-hospital death, femoral artery cannulation, left and right carotid artery dissection, aortic root replacement, aortic valve replacement, and early reintervention for bleeding. 

The incidence of ischemic stroke in this study was 27.5%. This is in accordance with previous studies that reported an incidence of up to 32.8% in NC [2,16,17,18,19]. Despite advances in brain protection methods, this is still a high incidence. Out of 89 patients with new postoperative ischemic stroke, 28 (21.8%) patients died in the intensive care unit, confirming the high in-hospital mortality previously reported [19,20,21]. Out of 223 patients with a BMI over 30 kg/m^2^, 75.7% had selective ACP, and 66 (29.6%) patients developed ischemic stroke. Data from the literature suggests that an increased risk of air or atheromatous embolism exists when the arch vessels are manipulated during surgery [22]. This could explain the high incidence of ischemic stroke in our study, considering the association between obesity and atherosclerosis. Inflammation is the link between the two and it is explained by the fact that adipose tissue releases adipokine, which in turn induces insulin resistance, endothelial dysfunction, and hypercoagulability, all of which promote atherosclerosis [23]. There are reports of longer circulatory arrest times when using BSACP because this technique is technically more complex, with an increased risk of stroke over 30 min [24,25]. Also, there are reports that operation times are higher in obese patients [26,27] and coupled with the technical difficulties of selective ACP institution could increase the rate of ischemic stroke. Operation times were not significantly longer in obese patients in our study (CPB time: 192.02 ± 73.18 vs. 211.39 ± 75.76; *p* = 0.558, aortic cross-clamp time: 113.53 ± 48.23 vs. 119.65 ± 45.86; *p* = 0.392, cerebral perfusion time: 31.58 ± 23.54 vs. 30.53 ± 24.78; *p* = 0.38). The mean cerebral perfusion time in our study was 30.8 ± 24.41 min and 30.53 ± 24.78 min in obese patients. Considering this, our data do not support the statement that BSACP is technically more complex. 

Other risk factors promoting atherosclerosis are smoking, hyperlipidemia, and diabetes. Smoking is a well-known factor in the development of cardiovascular disease via the initiation and progression of atherosclerosis. Mechanisms include oxidative stress induction, vascular inflammation, platelet coagulation, and vascular dysfunction [28]. Moreover, a study of 1320 autopsied specimens revealed atherosclerotic involvement of the aorta and coronary arteries in heavy smokers compared to nonsmokers [29]. The relationship between smoking and obesity is controversial. Some studies report no association between active smoking and BMI [30], while others found an association between smoking and lower BMI [30] and smoking cessation with increased BMI [31]. However, the association of smoking and obesity defined by BMI is described as a reverse causation, due to obese patients trying to lose weight by starting smoking [32]. Several studies report an association of smoking with obesity defined by waist circumference [33,34]. In our study, 29.1% of total patients and 22.2% of patients who developed ischemic stroke after surgery were active smokers. There was a significant difference between obese and non-obese patients regarding the frequency of smokers (*p* = 0.036), with 26% of obese patients being smokers. Smoking-induced atheromatosis at the level of the aortic arch could contribute to the high incidence of stroke in our study.

Atherosclerosis is a well-known complication of diabetes. It is estimated that 90% of patients with type 2 diabetes are attributable to obesity [35]. Common mechanisms include endothelial activation and inflammation, mitochondrial oxidative stress, changes in the extracellular matrix, and disruption of cellular defense systems [36]. One study reports a “common soil” between the two conditions named “nuclear factor KB” [37]. This “master regulator” triggers either hyperglycemia-induced endothelial dysfunction, or various micro RNAs that favor atherosclerosis [37]. Inflammation induced by chronic hyperglycemia induces endothelium dysfunction that promotes atherosclerosis [38]. Particularly relevant to our study, The Insulin Resistance Atherosclerosis Study (IRAS) reported that there is an association between increased atherosclerosis of the internal carotid artery with diabetes [39]. In our study, there were no significant differences between obese and non-obese patients regarding the incidence of diabetes, and only 7.9% of patients had diabetes. However, 30.4% of patients who developed stroke after surgery had diabetes and the increased carotid atherosclerosis described in these patients could contribute to the risk of stroke. 

Hyperlipidemia is another well-known factor for atherosclerosis and its association with obesity is well described [40,41]. A higher level of cholesterol is an independent risk factor for atherosclerosis of the carotid artery. Patients without any plaque had a significantly lower level of cholesterol compared with those with any degree of internal carotid artery stenosis [42]. Moreover, patients with untreated hyperlipidemia had more severe carotid artery plaques and a higher risk of plaque rupture according to morphology studies [42]. In our study, the incidence of hyperlipidemia was 69.5% and there was a significant difference between obese and non-obese patients regarding the incidence of hyperlipidemia (*p* = 0.023). Also, 79.2% of patients with stroke had hyperlipidemia. These results could suggest that hyperlipidemia contributes to atherosclerosis, especially at the level of the carotid arteries, and could raise the risk of stroke during aortic arch manipulation and selective cerebral perfusion. 

Obese patients with emergency surgery for ATAAD had a significantly higher in-hospital mortality (14.5% vs. 30.5%; *p* = 0.009). This finding is supported by data from several studies. Xiao Xu and al reported that morbid obesity is associated with an increased risk of in-hospital mortality in this group of patients [28]. Lio et al. reported a higher perioperative mortality rate and reduced 5-year survival rates in obese patients with emergency surgery for ATAAD [43]. Xiaogao et al. found that although obesity was more common in younger patients, it was more likely to cause death in elderly patients (age > 60 years) [13]. The high rate of mortality could be explained by the increased risk of low cardiac output syndrome and pulmonary complications in obese patients [43]. Postoperative acute lung injury is reported to have a higher incidence in obese patients and the risk of severe postoperative hypoxia seems to be of concern, especially in the female group [44,45]. Oxidative stress and an inflammatory response seem to be involved in the process of lung injury in obese patients [45].

In our study, aortic root replacement (11.6% vs. 26.9%, *p* = 0.009) and aortic valve replacement (5.8% vs. 20.2%; *p* = 0.005) had a higher frequency in obese patients. Several studies described an association between markers of obesity and the presence of an aortic aneurysm [14,46,47]. Inflammation caused by an imbalance of interleukin 6, 8 and TNF-alpha secreted by adipose tissue is thought to weaken and dilate vessels and may explain the role of obesity in aneurysm formation and weight loss may reduce the aortic diameter [15]. Obesity is also a risk factor for the development of calcified aortic stenosis and subsequent surgery. Two major studies including 71,817 patients from Sweden [48] and the Copenhagen General Population Study [49] support our findings. Also, obesity is a risk factor for calcified aortic stenosis progression [50,51]. Structural and metabolic changes are the link between obesity and calcified aortic stenosis. Obesity-related hypertension causes endothelial injury, while increased levels of atherogenic lipoproteins are deposited on the aortic leaflets, leading to calcification of the aortic valve [52,53].

Obesity may play a protective role in perioperative bleeding and transfusion needs [54]. Studies report this “obesity paradox” in patients undergoing myocardial revascularization [36,55]. Obese patients with ATAAD have better long-term survival compared to normal-weight patients and this could be explained by a younger age at dissection or a more typical presentation at admission [36,56,57]. Because of their increased body surface area and total blood volume, obese patients are less susceptible to hemodilution and CPB-related coagulopathy. Also, compression of mediastinal fat on minor bleeding sites could reduce the bleeding risk [54]. In our study, this does not seem to be the case, with obese patients having higher rates of early reintervention for bleeding (37.3% vs. 20.2%; *p* = 0.004). This could be explained by a higher incidence of coagulopathy when considering the complexity of ATAAD surgery, with HCA and longer CPB times, compared to myocardial revascularization alone or combined with valvular procedures. 

## 5. Conclusions

Data from 292 patients with emergency surgery for ATAAD were analyzed. More than 40 min of bilateral selective ACP in obese patients (BMI > 30 kg/m^2^) with emergency surgery for ATAAD was independently associated with a new postoperative ischemic stroke using multivariable analysis. Also, obese patients had a higher rate of early reintervention for bleeding, aortic root replacement, aortic valve replacement, femoral artery cannulation, left and right common carotid artery dissection, and higher hospital mortality. This result could be explained by the heavy atheromatous burden more frequent in obese patients at the level of the supra-aortic vessels, coupled with the risk of atheromatous embolism when selective bilateral ACP is used. When choosing a cerebral perfusion strategy in obese patients, this risk should be balanced with the fact that bilateral selective ACP is safer when HCA is more than 30 min, and a patient-tailored strategy should be considered. 

## Figures and Tables

**Table 1:** General characteristics of patients with emergency surgery for ATAAD; mean ± SD; *n* (%).

Preoperative	N = 292
Age (years)	59.42 ± 10.68
Euroscore	9.12 ± 1.63
Time from diagnosis to surgery (hours)	3.89 ± 2.37
Obesity (BMI > 30 kg/m^2^)	223 (76.4)
Hyperlipidemia	202 (69.5)
Smoke	85 (29.1)
Female gender	92 (31.5)
Hypertensive	269 (92.1)
Diabetes	23 (7.9)
COPD	14 (4.8)
Aortic diameter (mm)	6.36 ± 1.29
Family history of aortic dissection	7 (2.4)
Thoracic pain	269 (92.1)
Abdominal pain	36 (12.3)
Syncope	32 (11)
Lower leg ischemia	30 (10.3)
Mesenteric ischemia	5 (2)
Ischemic stroke	7 (2.4)
Cardiac tamponade	37 (12.7)
Cardiogenic shock	17 (5.8)
ST segment depression	32 (11)
ST segment elevation	25 (8.6)
Bicuspid aortic valve	27 (9.24)
Severe aortic regurgitation	74 (25.3)
Severe left ventricle dysfunction	14 (4.8)
Pericardial effusion	158 (54.1)
Intramural hematoma	25 (8.6)
Penetrating aortic ulcers	6 (2.1)
Ascending aorta intimal tear	142 (48.6)
Aortic arch intimal tear	13 (4.5)
Innominate artery dissection	55 (19.2)
Right common carotid artery dissection	22 (7.7)
Left common carotid artery dissection	37 (12.9)
Right subclavian artery dissection	13 (4.5)
Left subclavian artery dissection	24 (8.4)

**Table 2:** Intraoperative and postoperative data; mean ± SD; *n* (%).

	N = 292
Intraoperative	
Cardiopulmonary bypass time (min)	206.81 ± 75.48
Aortic cross clamp time (min)	118.2 ± 46.42
Cerebral perfusion time (min)	30.8 ± 24.41
Hypothermic circulatory arrest and cerebral perfusion	262 (90)
Axillary artery cannulation	144 (49.3)
Femoral artery cannulation	140 (47.9)
Ascending aorta cannulation	8 (2.74)
Aortic root replacement	68 (23.3)
Supracoronary ascending aorta replacement	283 (96.9)
Hemiarch replacement	134 (45.9)
Total arch replacement	16 (5.5)
Combined procedures	68 (23.28)
Aortic valve replacement	49 (16.8)
Coronary artery bypass grafting	16 (5.5)
Mitral valve repair	3 (1)
Femoro-femoral bypass	1 (0.34)
Coarctation repair	1 (0.34)
Peripheral V-A ECMO	2 (0.68)
Postoperative	
Early reintervention for bleeding	70 (24.1)
Mean intensive care stay (days)	11.2 ± 13.6
Mechanical ventilation over 24 h	216 (74)
Dialysis	149 (51)
Ischemic stroke	81 (27.5)
Hemiplegia	63 (21.57)
Coma	11 (3.76)
Aphasia	5 (2.6)
Visual field deficits	2 (0.68)
Mesenteric ischemia	22 (7.5)
Deep sternal wound infection	6 (2.1)

V-A ECMO: veno-arterial extracorporeal membrane oxygenator.

**Table 3:** Comparison between the patients with type A aortic dissection with and without obesity; mean ± SD; *n* (%).

	BMI < 30 kg/m^2^ *n* = 69	BMI > 30 kg/m^2^ *n* = 223	*p*
Preoperative			
Age	59.46 ± 8.16	59.41 ± 11.36	0.828
Smoke	27 (39.1)	56 (26)	0.036
Female sex	27 (39.1)	65 (29.1)	0.119
Hypertension	68 (98,6)	201 (90.1)	0.023
Hyperlipidemia	26 (37.7)	176 (79)	0.03
Diabetes	4 (5.8)	19 (8.5)	0.463
COPD	2 (2.9)	12 (5.4)	0.399
Family history of aortic dissection	2 (2.9)	5 (2.2)	0.755
Thoracic pain	65 (94.2)	204 (91.5)	0.463
Abdominal pain	8 (11.6)	28 (12.6)	0.832
Syncope	12 (17.4)	20 (9)	0.05
Lower limb ischemia	6 (8.7)	24 (10.8)	0.621
Mesenteric ischemia	1 (1.7)	5 (2.1)	0.825
Stroke	1 (1.4)	6 (2.7)	0.556
Cardiac tamponade	9 (13)	28 (12.6)	0.915
Cardiogenic shock	3 (4.3)	14 (6.3)	0.550
ST depression	10 (14.5)	22 (9.9)	0.282
ST elevation	7 (10.1)	18 (8.1)	0.591
Aortic regurgitation (TTE)	65 (94.2)	190 (85.2)	0.05
Pericardial effusion (TTE)	32 (46.4)	126 (56.5)	0.14
Severe left ventricular disfunction (TTE)	1 (1.4)	13 (5.8)	0.137
Ascending aorta Intimal flap (TEE)	8 (11.9)	50 (22.4)	0.06
Intramural hematoma (TEE)	4 (6)	21 (9.4)	0.378
Aortic plaque (TEE)	2 (3)	4 (1.8)	0.536
Innominate artery dissection (ADU)	34 (25)	39 (17.5)	0.178
Right common carotid artery dissection (ADU)	10 (14.1)	13 (5.8)	0.029
Left common carotid artery dissection (ADU)	18 (26.6)	20 (9)	<0.001
Right subclavian artery dissection	4 (6.2)	9 (4)	0.453
Left subclavian artery dissection	9 (12.5)	16 (7.2)	0.175
Intraoperative			
Cardiopulmonary bypass time (min)	192.02 ± 73.18	211.39 ± 75.76	0.558
Aortic cross clamp time (min)	113.53 ± 48.23	119.65 ± 45.86	0.392
Cerebral perfusion time (min)	31.58 ± 23.54	30.53 ± 24.78	0.38
Femoral artery cannulation	25 (36.2)	115 (51.6)	0.026
Axillary artery cannulation	41 (59.4)	103 (46.2)	0.055
Cerebral perfusion	45 (65.2)	145 (64.9)	0.957
Aortic root replacement	8 (11.6)	154 (26.9)	0.009
Ascending aorta replacement	64 (92.8)	219 (98.2)	0.022
Hemiarch replacement	32 (46.4)	102 (45.7)	0.926
Aortic arch replacement	2 (2.9)	14 (6.3)	0.281
Aortic valve replacement	4 (5.8)	45 (20.2)	0.005
Coronary artery bypass grafting	5 (7.2)	11 (4.9)	0.461
Mitral valve repair	0(0)	3 (1.3)	0.333
Early reintervention for bleeding	26 (37.3)	45 (20.2)	0.004
Postoperative			
Days in intensive care	13.18 ± 14.41	10.62 ± 13.37	0.859
In hospital death	10 (14.5)	68 (30.5)	0.009
Mechanical ventilation more than 24 h	56 (81.2)	160 (71.7)	0.12
Dialysis	30 (43.5)	119 (53.4)	0.151
Multiple system organ failure	17 (24.6)	81 (36.3)	0.072
Stroke	15 (21.7)	66 (29.6)	0.203
Mesenteric ischemia	6 (8.7)	16 (7.2)	0.676
Mediastinitis	1 (1.4)	5 (2.2)	0.685

ADU: arterial Doppler ultrasound; TTE: transthoracic echocardiography, TEE: transesophageal echocardiography.

**Table 4:** Comparison between the patients with type A aortic dissection with and without ischemic stroke; mean ± SD; *n* (%).

	Stroke + *n* = 81	Stroke − *n* = 211	*p*
Cardiopulmonary bypass time (min)	222.62 ± 70.8	200.75 ± 76.51	0.018
Aortic cross clamp time (min)	124.53 ± 48.3	115.782 ± 45.56	0.107
Cerebral perfusion time (min)	38.5 ± 28.9	27.92 ± 21.9	0.018
Cerebral perfusion pressure	65 ± 1.3	66 ± 2.1	0.761
Cerebral perfusion flow	190 ± 12.76	210 ± 5.39	0.271
Femoral artery cannulation	40 (49.4)	100 (47.4)	0.761
Axillary artery cannulation	39 (48.1)	105 (49.8)	0.805

**Table 5:** Modified Rankin Scale (mRS) at discharge.

mRS	N = 81
0	0 (0)
1	2 (1.62)
2	7 (5.67)
3	11 (8.91)
4	18 (14.58)
5	11 (8.91)
6	32 (25.92)

**Table 6:** Factors associated with ischemic stroke (multivariable statistics).

	OR	95%CI	*p*	OR	95%CI	*p*
Family history of aortic dissection	6.87	1.31–36.18	0.023			
Lower leg ischemia	0.07	0.01–0.58	0.013			
Tamponade at admission	4.87	2.37–9.96	<0.001	6.27	2.57–15.25	<0.001
Ascending aorta entry tear	0.17	0.05–0.56	0.004			
Innominate artery dissection	0.26	0.11–0.65	0.004	0.21	0.06–0.72	0.013
Left common subclavian artery dissection	0.2	0.06–0.68	0.01			
CPB time	1.004	1.001–1.007	0.028			
Cerebral perfusion time	0.01	0.005–0.031	0.008			
Aortic root replacement	0.42	0.21–0.85	0.017			
Hemiarch replacement	3.038	1.78–5.18	<0.001			
Reintervention for bleeding	1.93	1.09–3.41	<0.001			
BMI over 30 kg/m^2^ and BSACP over 40 min	2.18	1.12–4.22	0.021	2.35	1.36–4.86	0.021

**Table 7:** Risk factors associated with neurological complications [14,15].

	Risk Factor	OR	95% CI	*p*
German registry of acute aortic dissection [14]	Total surgery time	1.002	1.001–1.003	0.0001
	CPB time	1.002	1.001–1.004	0.0005
	CA time	1.009	1.003–1.015	0.0017
	Malperfusion of 3 or more organs	2.206	1.278–3.810	0.038
Nordic consortium of acute aortic disection [15]	CBP time	1.19	1.11–1.26	<0.001
	Cerebral ischemia	4.28	2.56–7.17	<0.001
	Tamponade	1.85	1.12–3.05	0.015
	Cardiogenic shock	2.45	1.20–4.98	0.013

## Data Availability

The data presented in this study are available on reasonable request from the corresponding author.
